# The Neuro–Cardio–Renal Stress Index (NCR-SI): A Pragmatic Composite Framework for Characterizing Multisystem Burden in Multimorbid Patients

**DOI:** 10.3390/diagnostics16081120

**Published:** 2026-04-08

**Authors:** Ana Trandafir, Oceane Colasse, Marc Cristian Ghitea, Evelin Claudia Ghitea, Timea Claudia Ghitea, Roxana Daniela Brata, Alexandru Daniel Jurca

**Affiliations:** 1 Doctoral School of Biological and Biomedical Sciences, University of Oradea, 1 University Street, 410087 Oradea, Romania; trandafirana12@yahoo.com; 2 Faculty of Medicine and Pharmacy, University of Oradea, 1st Decembrie Street, 410073 Oradea, Romania; colasse.oceane@student.uoradea.ro (O.C.); ghitea.marccristian@student.uoradea.ro (M.C.G.); ghitea.evelinclaudia@student.uoradea.ro (E.C.G.); 3 Pharmacy Department, Faculty of Medicine and Pharmacy, University of Oradea, 1 University Street, 410087 Oradea, Romania; 4 Department of Medical Disciplines, Faculty of Medicine and Pharmacy, University of Oradea, 1 University Street, 410087 Oradea, Romania; alexjurca@uoradea.ro

**Keywords:** anxiety disorders, chronic kidney disease, insulin resistance, multimorbidity, psychiatric comorbidity, reno-metabolic risk, Charlson Comorbidity Index, electronic medical records, composite index

## Abstract

**Background**: Multimorbidity frequently involves overlapping neuro-psychic, cardiometabolic, and renal disturbances, yet clinical assessment often relies on diagnosis-based comorbidity counts that may not fully capture cumulative physiological stress. We developed the Neuro–Cardio–Renal Stress Index (NCR-SI) as a pragmatic composite framework to describe multisystem burden using routinely available clinical data. **Methods**: This cross-sectional study analyzed electronic medical record data from adult patients with chronic conditions. NCR-SI integrates three domains: neuro-psychic burden (text-derived indicators and psychotropic medication use), cardiometabolic stress (triglyceride–glucose index and cardiometabolic diagnoses), and renal function (MDRD-estimated eGFR staging). Importantly, this study is not intended to demonstrate incremental predictive value over individual components or established comorbidity indices. Rather, it presents NCR-SI as a transparent, domain-based descriptive framework and reports its internal coherence and distribution across clinically recognizable multimorbidity contexts. **Results**: A total of 148 patient records were screened; 143 patients met complete-case criteria and were included in the main NCR-SI analyses. NCR-SI ranged from 0 to 10 (median 5). Higher scores were observed in renometabolic profiles. NCR-SI showed expected structural associations with declining renal function (eGFR; ρ ≈ −0.71), moderately with the TyG index (ρ ≈ 0.42), and weakly with medication burden. Correlation with age-adjusted CCI was minimal (ρ ≈ 0.09), indicating limited overlap with diagnosis-based comorbidity counts. Domain-specific correlations were consistent with predefined score construction rules, particularly between the renal domain and eGFR, and between the cardiometabolic domain and TyG. **Conclusions**: NCR-SI provides a pragmatic, integrative descriptor of neuro-cardio-renal stress using routinely collected clinical data. Rather than replacing established comorbidity indices, NCR-SI may complement them by summarizing multidimensional physiological burden patterns. NCR-SI is proposed as a research-oriented, hypothesis-generating descriptive framework. External validation in independent cohorts and longitudinal evaluation against clinically meaningful outcomes (e.g., hospitalization, mortality, functional status, healthcare utilization) are required before any claims of clinical performance can be made.

## 1. Introduction

Multimorbidity has become a defining feature of contemporary clinical medicine, particularly in aging populations where cardiometabolic disorders, chronic kidney disease (CKD), and psychological distress frequently coexist. These conditions rarely occur in isolation and often interact through shared mechanisms such as systemic inflammation, neuroendocrine dysregulation, endothelial dysfunction, and maladaptive health behaviors. As a result, patient burden increasingly reflects the cumulative effect of multiple interacting systems rather than single-organ pathology [1,2,3,4].

Cardiometabolic diseases—including hypertension, dyslipidemia, insulin resistance, obesity, and type 2 diabetes—remain a major contributor to global morbidity. CKD, often described as a “silent pandemic,” commonly coexists with cardiometabolic abnormalities and further amplifies cardiovascular and metabolic vulnerability. In parallel, anxiety, depression, sleep disturbances, and psychosomatic symptoms are highly prevalent among patients with chronic disease and are associated with reduced adherence, impaired quality of life, and increased healthcare utilization [5,6,7,8,9].

Several indices have been developed to quantify comorbidity burden, most notably the Charlson Comorbidity Index (CCI), which is widely used for mortality risk adjustment. However, traditional comorbidity indices are primarily diagnosis-based and were designed for prognostic stratification rather than for describing multidimensional physiological stress. They typically do not incorporate metabolic stress markers or structured indicators of neuro-psychic burden. Consequently, they may not fully capture the integrative, cross-system burden observed in everyday multimorbid patients [10,11,12].

Beyond diagnosis-based indices such as the Charlson Comorbidity Index, several prior approaches have attempted to characterize multisystem burden using physiology-oriented constructs, including metabolic risk scores, cardiometabolic stress indices, and psychosocial burden frameworks. However, few models explicitly integrate renal function, metabolic stress, and neuro-psychic burden into a unified descriptive framework using routinely available EMR-derived parameters.

Renal function represents a central node within cardiometabolic multimorbidity. Estimated glomerular filtration rate (eGFR), particularly when derived using the MDRD equation, is a standardized and guideline-aligned measure strongly associated with cardiovascular risk, metabolic syndrome, and polypharmacy burden. Similarly, the triglyceride–glucose (TyG) index has emerged as a pragmatic surrogate marker of insulin resistance and metabolic stress. Together, these routinely available measures reflect systemic vulnerability [13,14,15].

At the same time, psychological and psychosomatic burden is increasingly recognized as a parallel dimension of multimorbidity. Although often documented in electronic medical records (EMR) through descriptive notes or treatment patterns rather than standardized scales, these data still reflect clinically recognized neuro-psychic involvement in real-world practice [16,17,18,19].

In this context, there is value in exploratory frameworks that integrate renal, metabolic, and neuro-psychic dimensions into a single descriptive construct using routinely available clinical data. Such frameworks are not intended to replace established prognostic indices but to provide complementary summaries of patient complexity.

The present study introduces the Neuro–Cardio–Renal Stress Index (NCR-SI), a descriptive composite index integrating the following:

- neuro-psychic burden documented in EMR,
- cardiometabolic stress reflected by the TyG index and metabolic diagnoses, and
- renal dysfunction quantified by MDRD-estimated eGFR [20,21].

Rather than serving as a diagnostic or prognostic tool, NCR-SI is proposed as a research-oriented, hypothesis-generating framework for summarizing the burden of multimorbidity across interacting systems. For contextual benchmarking, a Charlson Comorbidity Index (CCI) was additionally derived to position NCR-SI relative to an established comorbidity measure.

The objectives of this study were as follows:

1. to describe the distribution of NCR-SI across clinically recognizable multimorbidity contexts,
2. to explore the internal correlation structure of its domains, and
3. to examine how NCR-SI relates to a conventional comorbidity index (CCI) in a real-world EMR dataset.

While several established indices quantify diagnosis-based comorbidity burden, fewer approaches attempt to summarize cross-system physiological stress using routinely available functional and metabolic indicators. In clinical practice, multimorbidity often manifests through interacting renal, metabolic, and psychosocial disturbances that may not be adequately captured by diagnosis counts alone. The present exploratory framework was therefore developed to examine whether a transparent aggregation of such routinely documented parameters could provide complementary descriptive insight into multisystem burden patterns.

## 2. Materials and Methods

### 2.1. Study Design and Population

This cross-sectional observational study used routinely collected data from a single-center institutional EMR system. The source population consisted of adult patients (≥18 years) with documented chronic conditions who had at least one clinical encounter within the study extraction window (see data extraction procedures). Patients were included if the minimum data required to compute all NCR-SI domains were available (TyG-related labs, creatinine for eGFR, and a diagnostic text field). A complete-case approach was used for NCR-SI computation; therefore, selection bias due to laboratory availability is possible, and generalizability may be restricted to settings with comparable testing and documentation practices.

Inclusion criteria required the availability of the following:

- fasting glucose and triglycerides for TyG calculation,
- serum creatinine for MDRD-based eGFR estimation,
- a diagnostic text field allowing neuro-psychic screening.

Patients with missing key laboratory or diagnostic data preventing the computation of any NCR-SI domain were excluded. A complete-case approach was applied for NCR-SI calculation.

### 2.2. Data Collection and Variables

Demographic variables included age and sex. Extracted clinical and laboratory variables comprised the following:

- Renal variables
- Serum creatinine
- MDRD-estimated eGFR
- Urinary albumin-to-creatinine ratio (UACR, when available)
- Cardiometabolic variables
- Fasting glucose
- Triglycerides
- TyG index
- Number of chronic medications
- Diagnostic information

Free-text diagnostic entries and medication records were used for rule-based phenotyping.

#### Group Definition

Patients were categorized into three descriptive multimorbidity contexts based on EMR-derived diagnoses and renal function:

(1) Cardiometabolic: presence of ≥1 cardiometabolic condition (hypertension, type 2 diabetes, dyslipidemia, obesity/metabolic syndrome) with preserved renal function (eGFR ≥ 60 mL/min/1.73 m^2^).
(2) Renometabolic: presence of cardiometabolic condition(s) and reduced renal function (eGFR < 60 mL/min/1.73 m^2^) consistent with CKD staging.
(3) Other comorbidities: chronic conditions not meeting criteria for (1) or (2); this small heterogeneous group was used as a descriptive comparator only.

### 2.3. Definition and Construction of the Neuro–Cardio–Renal Stress Index (NCR-SI)

NCR-SI was conceptualized as a composite descriptor of cumulative physiological stress across neuro-psychic, metabolic, and renal domains. Domain weights were chosen to maintain comparable contributions from each system while allowing greater granularity for renal impairment, given its staged clinical classification.

Each domain contributed additively to the total NCR-SI score. The neuro-psychic and cardiometabolic domains were scored from 0 to 3 points each, while the renal domain was scored from 0 to 4 points, resulting in a total possible range of 0–10 points. Domains were not statistically weighted but structurally balanced to ensure comparable contribution across systems, with slightly greater granularity allocated to the renal domain because renal dysfunction is clinically categorized through internationally standardized eGFR staging systems, reflects progressive multisystem vulnerability, and occupies a central position within cardio–reno–metabolic multimorbidity frameworks. Thresholds for TyG and eGFR were predefined based on established clinical categories and literature-supported cut-offs rather than derived from the present dataset.

The scoring structure was selected a priori to prioritize interpretability and clinical transparency rather than statistical optimization. No regression-based weighting, latent variable modeling, or data-driven calibration was performed in this initial framework study, and future work should evaluate alternative weighting schemes and empirical refinement in independent cohorts.

#### 2.3.1. Neuro-Psychic Domain (0–3 Points)

Neuro-psychic burden was operationalized as documented psychological or psychosomatic involvement in routine care rather than as a formal psychiatric diagnosis.

Keyword dictionaries were developed based on commonly documented terms in routine EMR practice, including anxiety-related, depressive, sleep-related, psychosomatic, and cognitive symptom terminology. Preprocessing included text normalization, lowercasing, and removal of diacritics. Because the approach was rule-based and dependent on clinician documentation patterns, both under-classification and misclassification remain possible.

A rule-based text-mining approach was applied to normalized diagnostic text (lowercased, diacritics removed). Keyword dictionaries included terms related to the following:

- anxiety spectrum
- depressive symptoms
- sleep disturbances
- psychosomatic/stress-related complaints
- cognitive symptoms
- severe psychiatric conditions

Psychotropic medication records (anxiolytics, antidepressants, antipsychotics) were used as complementary indicators.

Scoring reflected the accumulation of documented indicators, not symptom severity.

#### 2.3.2. Cardiometabolic Domain (0–3 Points)

Metabolic stress was quantified using the TyG index:

TyG = ln [triglycerides (mg/dL) × glucose (mg/dL)/2]

Points were assigned based on established TyG ranges reflecting insulin resistance gradients. An additional point was assigned for documented cardiometabolic diagnoses (hypertension, diabetes, dyslipidemia, obesity, or metabolic syndrome).

#### 2.3.3. Renal Domain (0–4 Points)

Renal burden was based on MDRD-estimated eGFR staging, aligned with CKD severity categories. Greater weight was allocated to renal impairment due to its strong systemic implications and widely accepted staging framework.

UACR was analyzed descriptively but not incorporated into point allocation.

### 2.4. Charlson Comorbidity Index (CCI)

For contextual benchmarking, a Charlson Comorbidity Index (CCI) was derived using a rule-based mapping of free-text diagnoses combined with objective renal classification.

Charlson categories identifiable in text (e.g., heart failure, cerebrovascular disease, dementia, chronic pulmonary disease, diabetes, malignancy) were flagged using predefined keyword sets. Renal disease was defined using eGFR < 60 mL/min/1.73 m^2^ or documented dialysis.

Age-adjusted CCI was subsequently calculated according to the original Charlson framework. Given the absence of ICD coding, this approach is considered a text-derived CCI proxy and is interpreted as a contextual comparator rather than a fully validated administrative CCI.

### 2.5. Statistical Analysis

All analyses were conducted using SPSS (v30) and Python (v3.11, using pandas and SciPy libraries). Statistical analyses were designed to explore the internal structure, descriptive distribution, and construct coherence of the Neuro–Cardio–Renal Stress Index (NCR-SI), rather than to evaluate predictive or discriminatory performance.

Given non-normal distributions, nonparametric methods were applied. Continuous variables are reported as medians and interquartile ranges.

Spearman correlations were used to explore the following:

- associations between NCR-SI and biomarkers,
- inter-domain relationships,
- associations between NCR-SI and CCI.

Group comparisons used Kruskal–Wallis tests. These analyses were interpreted descriptively.

Validation status: The rule-based neuro-psychic phenotyping strategy was implemented as a pragmatic EMR proxy and was not formally validated against structured psychiatric diagnoses or standardized symptom scales in this dataset. Therefore, sensitivity, specificity, and agreement metrics could not be estimated in the current study and should be addressed in future work using annotated clinical notes or structured diagnostic references.

No analyses of discrimination, calibration, incremental validity, or outcome prediction were performed, as the dataset was cross-sectional and not designed for prognostic modeling.

Additional weighting sensitivity analysis

As the renal domain contributes a maximum of 4 points, whereas the neuro-psychic and cardiometabolic domains contribute up to 3 points each, an additional exploratory sensitivity analysis was conducted to evaluate the impact of alternative domain weighting schemes.

Two symmetric formulations were examined:

(1) equal weighting of all domains (0–3/0–3/0–3; total range 0–9), and

(2) expanded symmetric weighting (0–4/0–4/0–4; total range 0–12).

For each formulation, domain variance contributions, correlation structures, and group-level score distributions were compared descriptively with the original NCR-SI structure. These analyses were intended to assess whether the observed score behavior was driven primarily by renal weighting asymmetry.

In addition, exploratory weighting sensitivity analyses were conducted to assess the impact of alternative domain scoring structures on overall index behavior.

### 2.6. Ethical Considerations

The study was conducted in accordance with the Declaration of Helsinki and approved by the Institutional Ethics Committee of the University of Oradea (protocol No. 46/31 October 2025). This was a retrospective analysis of routinely collected EMR data. Patients provided general consent at admission for the use of anonymized clinical data for research purposes. All data were processed in a de-identified format for analysis.

## 3. Results

### 3.1. Demographic Characteristics of the Three Groups

A total of 148 patient records were screened, of which 143 met complete-case criteria for NCR-SI computation. Exclusions were mainly due to missing laboratory data required for TyG or eGFR calculation.

The cohort included the following:

- 48 cardiometabolic patients
- 82 renometabolic patients
- 13 patients with other chronic conditions

Median age and medication burden increased progressively from cardiometabolic to renometabolic contexts, consistent with higher clinical complexity.

As inclusion required the availability of key laboratory parameters and diagnostic text, the analyzed cohort may overrepresent patients undergoing more intensive testing.

As expected from the cohort definition, eGFR was lowest in the renometabolic group, while TyG values were elevated in both cardiometabolic and renometabolic patients.

Together, these demographic findings are consistent with the cohort classification strategy and illustrate progressive metabolic–renal complexity across groups. These baseline differences support subsequent analyses of biochemical profiles, psychiatric burden, and NCR-SI distribution across the three multimorbidity clusters.

The range reported in preliminary summaries reflects the total screened cohort (*n* = 148). Meanwhile, all correlation and score-based analyses were conducted in the complete-case sample (*n* = 143).

### 3.2. NCR-SI Distribution Across Clinical Contexts

NCR-SI demonstrated a graded distribution across multimorbidity contexts. Median scores were highest in the renometabolic group, intermediate in cardiometabolic patients, and lowest in the heterogeneous chronic disease group.

Renometabolic patients concentrated the largest proportion of moderate-to-high NCR-SI categories, reflecting accumulation of renal and metabolic burden. Cardiometabolic patients clustered predominantly in the moderate range, whereas most individuals in the heterogeneous group fell into the low-stress category.

**Figure 1:**
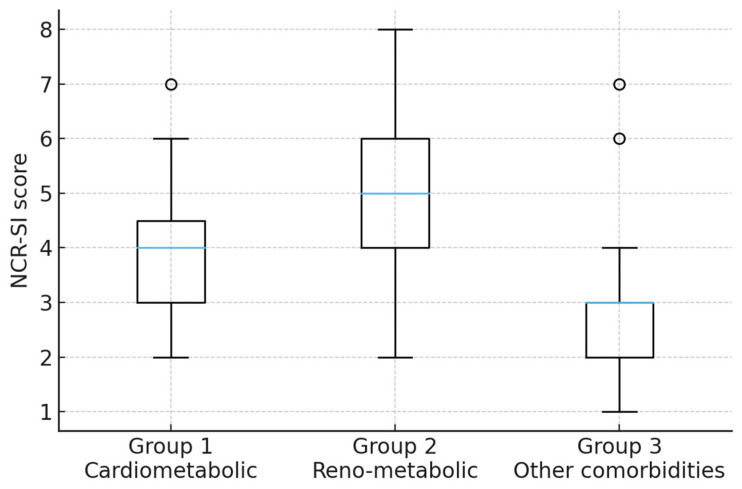
Distribution of Neuro–Cardio–Renal Stress Index (NCR-SI) scores across the three multimorbidity groups. Boxplots display the median, interquartile range, and range of NCR-SI values, with outliers shown as individual points. NCR-SI was calculated as the sum of neuro-psychic, cardiometabolic, and renal domains, with renal function assessed using MDRD-estimated eGFR. The figure illustrates group-level differences in cumulative stress burden and does not imply causal relationships. The blue horizontal line within each box represents the median value, the box boundaries indicate the interquartile range (IQR; 25th–75th percentile), and the whiskers denote the minimum and maximum values excluding outliers. Open circles indicate outlier values.

These patterns illustrate how cumulative neuro-cardio-renal stress varies across common multimorbidity profiles (Table 1 and Figure 1).

### 3.3. NCR-SI Risk Categories

To further characterize the clinical relevance of the Neuro–Cardio–Renal Stress Index (NCR-SI), patients were stratified into low (0–3), moderate (4–6), and high (7–10) stress categories based on their total NCR-SI scores. The distribution of these risk categories across the three multimorbidity groups is presented in Table 2.

NCR-SI ranged from 0 to 10 points, with a median value of 5 (IQR 4–6). Higher scores were predominantly observed in renometabolic patients, whereas lower values clustered in heterogeneous chronic conditions.

In Group 1, most patients were classified as moderate (63.8%), with few high-score cases (2.1%). Group 2 showed a right-shifted distribution, with 11.1% in the high category, consistent with combined renal and metabolic burden. Group 3 was small and heterogeneous and is reported descriptively only.

The renometabolic group (Group 2) exhibited the highest overall stress burden, with 69.1% of patients classified as moderate risk and 11.1% reaching the high NCR-SI category. Only 19.8% of patients in this group were classified as low risk.

In contrast, Group 3, given its small size and heterogeneity, is interpreted strictly as a descriptive comparator, and no inferential conclusions are drawn from this subgroup. The majority of patients (76.9%) were classified as low risk, while 15.4% were moderate risk and 7.7% were high risk. Although this group was small and heterogeneous, the presence of high-risk cases highlights that elevated neuro–cardio–renal stress may also occur outside classical cardiometabolic or renometabolic phenotypes.

The near-zero correlations between the neuro-psychic domain and biochemical markers should be interpreted cautiously. Such findings may reflect partial conceptual independence of psychological burden from laboratory parameters, but they may also indicate limited measurement sensitivity of the proxy indicators used in this study.

Because the neuro-psychic component was derived from rule-based text phenotyping and medication records, its construct validity and diagnostic accuracy remain uncertain.

Overall, the categorical distribution of NCR-SI scores demonstrates a graded and clinically coherent stratification of multimorbidity burden across the three groups. The renometabolic cluster concentrated the highest proportion of moderate-to-high stress profiles, whereas cardiometabolic patients primarily occupied intermediate risk levels, and patients with other comorbidities were predominantly classified as low risk. These patterns illustrate how cumulative neuro-cardio-renal stress is distributed across the three multimorbidity contexts within this dataset.

### 3.4. Weighting Sensitivity Analysis

Under the original asymmetric weighting structure, the renal domain accounted for approximately 45.8% of total score variance, compared with 28.6% and 25.6% for the cardiometabolic and neuro-psychic domains, respectively.

Under the 0–3/0–3/0–3 symmetric formulation, the relative renal contribution decreased to 34.1%, while cardiometabolic and neuro-psychic contributions increased to 32.8% and 33.1%, respectively.

Similarly, the correlation between total NCR-SI and the renal domain decreased from ρ = 0.755 in the original structure to ρ = 0.681 under equal weighting. In contrast, correlations with the cardiometabolic and neuro-psychic domains became more balanced (ρ = 0.601 and ρ = 0.587, respectively) (Table 3).

These findings indicate that the statistical contribution of the renal domain to the composite score decreases under symmetric weighting schemes, suggesting that part of its influence in the original NCR-SI formulation is structurally determined.

Correlation coefficients between the total score and individual domains became more balanced in the symmetric models, suggesting that part of the stronger association observed in the original NCR-SI structure was attributable to differential point allocation rather than intrinsic clinical dominance of renal dysfunction.

Overall, the descriptive behavior of the index appears partially influenced by domain weighting choices.

### 3.5. Comparison with Charlson Comorbidity Index

To contextualize NCR-SI relative to an established comorbidity metric, a text-derived Charlson Comorbidity Index (CCI), including an age-adjusted version, was computed.

Spearman correlation analysis showed a strong association between CCI and age-adjusted CCI (ρ = 0.794, *p* < 0.001), as expected.

In contrast, NCR-SI demonstrated no significant correlation with age-adjusted CCI (ρ = 0.085, *p* = 0.311) and only a weak correlation with the non-age-adjusted CCI (ρ = 0.172, *p* = 0.040).

These findings indicate limited overlap between NCR-SI and traditional diagnosis-based comorbidity indices, suggesting that NCR-SI captures dimensions of patient burden not fully represented by comorbidity counts alone (Table 4).

**Figure 2:**
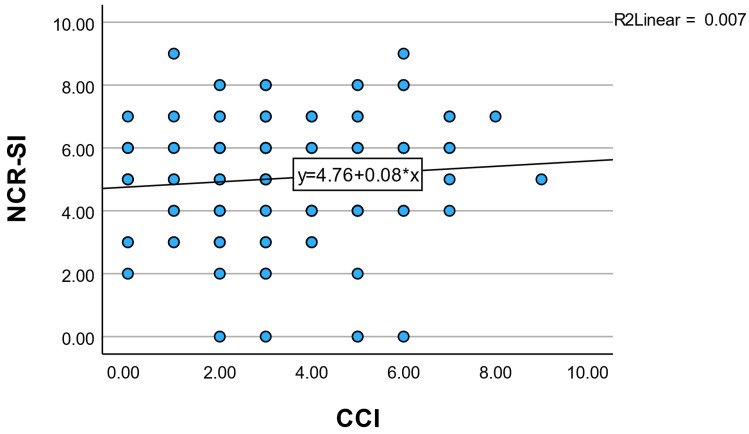
Scatter plot showing the relationship between the Neuro–Cardio–Renal Stress Index (NCR-SI) and the age-adjusted Charlson Comorbidity Index (CCI). Each point represents one patient. The fitted trend line indicates a weak positive association between the two indices, with wide dispersion of NCR-SI values across CCI levels. The solid line indicates the linear regression fit $\left(y=4.76+0.08x\right)$, with a coefficient of determination $R^{2}=0.007$, suggesting no meaningful linear correlation.

Dispersion of NCR-SI values within similar CCI ranges further suggests that patients with comparable diagnosis-based comorbidity burden may differ in cumulative neuro-cardio-renal stress. This supports the interpretation of NCR-SI as a complementary descriptor rather than a substitute for traditional comorbidity indices (Figure 2). The observed association should be interpreted as a descriptive structural comparison and not as evidence of external validation.

### 3.6. Correlation Structure Between NCR-SI, Its Domains, and Clinical Biomarkers

Spearman correlation analysis was performed to examine the internal structure of the Neuro–Cardio–Renal Stress Index (NCR-SI) and its associations with renal, metabolic, and neuro-psychic biomarkers. Correlation coefficients between the total NCR-SI score and its individual domains (N, C, R), as well as key clinical variables, are summarized in Table 4.

As expected from the score construction, the total NCR-SI showed moderate-to-strong positive correlations with its component domains, with the strongest association observed for the renal domain (R; ρ = 0.755), followed by the cardiometabolic domain (C; ρ = 0.533) and the neuro-psychic domain (N; ρ = 0.509). This pattern indicates that all three domains contribute meaningfully to the composite score while reflecting different magnitudes of influence within the integrated structure.

The renal domain demonstrated a very strong inverse correlation with MDRD-estimated eGFR (ρ = –0.973) and a weak-to-moderate positive correlation with UACR (ρ = 0.207). Similarly, the total NCR-SI score correlated inversely with eGFR (ρ = –0.709) and showed a weak positive correlation with UACR (ρ = 0.202). These correlations primarily confirm correct operational implementation of domain scoring, since the same biomarkers are directly incorporated into point allocation. They should therefore not be interpreted as evidence of independent construct validity.

The cardiometabolic domain showed a strong positive correlation with the TyG index (ρ = 0.785), while the total NCR-SI also correlated moderately with TyG (ρ = 0.416). These findings indicate that metabolic stress, as captured by insulin resistance–related markers, contributes substantially to overall neuro–cardio–renal stress burden.

In contrast, the neuro-psychic domain exhibited minimal correlations with biochemical markers, including eGFR, UACR, and TyG (absolute ρ values close to zero). Despite this relative independence from laboratory parameters, the neuro-psychic domain maintained a moderate correlation with the total NCR-SI score (ρ = 0.509), suggesting that psychiatric and psychosomatic burden represents a parallel and additive dimension of multimorbidity rather than a direct reflection of biological severity.

**Figure 3:**
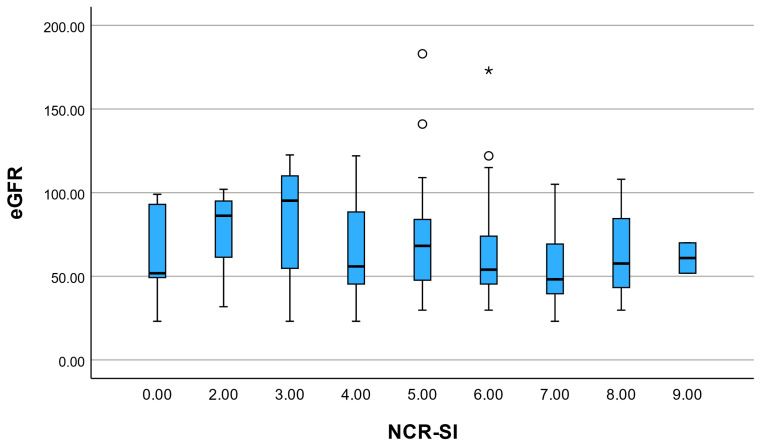
Distribution of estimated glomerular filtration rate (eGFR) across NCR-SI score levels. As eGFR contributes directly to renal domain scoring, this association reflects internal structural coherence rather than independent validation. Open circles represent mild outliers (values located 1.5–3 IQRs from the quartiles), while the asterisk indicates an extreme outlier (>3 IQRs from the quartiles).

NCR-SI showed a moderate positive correlation with the TyG index (ρ = 0.416, *p* < 0.001) and a strong negative correlation with eGFR (ρ = −0.709, *p* < 0.001). A weaker but significant association was observed with the number of chronic medications (ρ = 0.244, *p* = 0.003) (Figure 3). As renal function contributes directly to score construction, this association reflects internal score coherence rather than independent validation.

Importantly, inter-domain correlations between the neuro-psychic domain and the cardiometabolic or renal domains were weak (ρ ≤ 0.163), suggesting partial independence among NCR-SI components. This multidimensional correlation pattern supports the conceptual framework of NCR-SI as an integrative index that captures distinct yet complementary axes of multimorbidity, rather than redundant representations of the same underlying process (Table 5).

### 3.7. Sensitivity Analysis

In addition to weighting sensitivity testing, further exploratory analyses were performed to evaluate robustness to threshold variation within individual domains.

To evaluate the robustness of the Neuro–Cardio–Renal Stress Index (NCR-SI) construction, limited sensitivity analyses were performed focusing on the cardiometabolic and renal components of the score. These analyses were designed to assess whether the observed group-level differences and correlation structures were dependent on specific threshold definitions or scoring formulations.

First, the cardiometabolic domain was re-evaluated using shifted TyG thresholds (±0.2 units) to account for potential boundary effects in insulin resistance categorization. Under these alternative threshold definitions, the distribution of NCR-SI scores across the three multimorbidity groups and the relative ordering of the groups remained unchanged, and the correlation patterns between NCR-SI, the cardiometabolic domain, and TyG values were preserved.

Second, the renal domain, based on MDRD-estimated eGFR staging, was compared with the original renal scoring formulation used in the preliminary analyses. Despite minor differences in absolute point allocation at the individual level, the overall NCR-SI distribution, group-level contrasts, and correlation structure with renal biomarkers remained qualitatively consistent across formulations.

Together, these sensitivity analyses indicate that the descriptive stratification capacity and internal structure of NCR-SI are stable across reasonable variations in domain thresholds and renal scoring approaches. These exploratory analyses suggest that the descriptive distribution and internal correlation structure of NCR-SI were not materially altered by reasonable variations in domain thresholds.

## 4. Discussion

This study introduces and applies the Neuro–Cardio–Renal Stress Index (NCR-SI) as a descriptive, multidimensional framework for summarizing multimorbidity burden through an integrated neuro–cardio–renal stress construct across neuro-psychic, metabolic, and renal domains in a real-world clinical cohort.

The observed distribution of NCR-SI across clinically recognizable multimorbidity contexts illustrates how cumulative stress burden varies in routine care settings. These patterns should be interpreted as descriptive reflections of underlying clinical complexity, not as evidence of score performance or causal relationships [22,23,24,25,26].

Patients in the renometabolic group exhibited the highest NCR-SI values and the greatest proportion of moderate-to-high stress profiles. This pattern is consistent with the coexistence of impaired renal function and metabolic abnormalities captured by the score’s construction. The observed alignment between the renal domain and MDRD-estimated eGFR, as well as the association between NCR-SI and renal markers, should be interpreted as structural coherence of the index, rather than as confirmation of renal dysfunction as a causal determinant of multimorbidity. Within the cross-sectional design, these findings indicate that renal impairment contributes substantially to the composite score, as summarized by the NCR-SI, without implying directionality or temporal precedence [27,28,29,30,31].

The very weak correlation between NCR-SI and the age-adjusted CCI deserves particular emphasis. Conceptually, this limited overlap is consistent with the different constructs captured by the two indices. Whereas CCI quantifies diagnosis-based comorbidity burden primarily for mortality risk stratification, NCR-SI was designed to summarize cross-system physiological stress, including metabolic dysregulation and documented neuro-psychic involvement. Methodologically, the text-derived nature of the CCI proxy used in this study may also contribute to partial discordance. Together, these findings support the interpretation that NCR-SI is not a competing comorbidity index but rather a complementary framework that captures dimensions of burden not fully represented by diagnosis counts alone.

The neuro-psychic component should therefore be interpreted as a documentation-based signal of psychosocial involvement rather than as a validated measure of psychiatric morbidity. The cardiometabolic group displayed a distinct NCR-SI profile characterized by intermediate composite scores. Although renal function was relatively preserved in this group, metabolic stress and neuro-psychic indicators contributed to the overall burden. The moderate correlation between the neuro-psychic domain and the total NCR-SI, combined with minimal correlations between psychiatric indicators and biochemical markers, is consistent with co-occurrence patterns that documented neuro-psychic involvement, as captured in routine clinical records, represents an independent and complementary dimension of multimorbidity. These findings support the internal descriptive coherence of NCR-SI within a cross-sectional setting, without implying longitudinal stability or clinical predictive utility [32,33,34,35]. Associations between NCR-SI domains and the biomarkers used in their construction represent structural correlations rather than independent validation findings. Demonstration of external validity would require evaluation against outcomes not included in score derivation, such as hospitalization risk, functional decline, or disease progression.

The group with other comorbidities represented the smallest and most heterogeneous cluster, characterized by predominantly low NCR-SI scores and a limited number of moderate-to-high risk cases. Given its size and diversity, this group should be interpreted cautiously, primarily as a comparator that illustrates that elevated neuro–cardio–renal stress is not uniformly present across all chronic disease profiles. The presence of isolated high-risk cases underscores the heterogeneity of multimorbidity but does not allow generalizable inferences.

Correlation analyses and heatmap visualization further supported the multidimensional structure of NCR-SI. Each domain aligned primarily with its corresponding clinical markers, while maintaining partial independence from other domains. These correlations support the internal structural coherence of the NCR-SI domains, rather than external validation or causal inference. Importantly, the visualization and correlation results were used descriptively and were not intended to suggest biological mechanisms or causal relationships [36,37,38].

From a methodological perspective, this study contributes to the growing literature on multimorbidity assessment by proposing a pragmatic, clinically interpretable composite index that incorporates psychiatric vulnerability alongside metabolic and renal stress. Unlike traditional organ-specific scores, NCR-SI aims to reflect patient complexity as encountered in real-world settings, where psychological distress frequently coexists with somatic disease and influences overall burden, yet is not captured by standard biomedical indices [36,39,40,41,42,43,44].

### 4.1. Strengths and Limitations

Several strengths of this study warrant consideration. First, NCR-SI represents a novel integrative framework that combines neuro-psychic, cardiometabolic, and renal dimensions into a single composite score, addressing a recognized gap in multimorbidity assessment. Second, the comparative analysis across three clinically defined groups allowed structured evaluation of how composite stress burden varies across common multimorbidity patterns. Third, the use of routinely available clinical data enhances the potential applicability of NCR-SI in real-world settings.

However, important limitations must be acknowledged. The cross-sectional design precludes causal inference and does not allow assessment of temporal relationships among psychiatric, metabolic, and renal factors. The relatively small size of the heterogeneous comorbidity group limits statistical power and generalizability for this subgroup. Future studies incorporating validated psychiatric instruments are needed to refine this component.

In addition, some laboratory variables, including UACR, were not available for all patients and were therefore excluded from score construction and used descriptively. Although internal coherence of NCR-SI was supported by correlation analyses and sensitivity testing, external validation in independent cohorts and longitudinal evaluation of prognostic value remain essential next steps.

The complete-case design may have preferentially included patients undergoing more frequent biochemical testing, potentially enriching the cohort for individuals with greater clinical complexity and thereby influencing observed score distributions.

A major methodological limitation concerns the neuro-psychic domain. This component reflects documentation patterns in routine care rather than standardized psychiatric assessment. Consequently, NCR-SI neuro-psychic scores should be interpreted as indicators of clinically recognized distress rather than as validated measures of psychiatric burden.

The possibility that this domain under-captures or misclassifies neuro-psychic morbidity cannot be excluded.

Importantly, the present analysis is strictly descriptive and hypothesis-generating. The observed correlations between NCR-SI and its component biomarkers primarily reflect score construction and internal structural coherence rather than independent construct validation. No external validation, discrimination analysis, calibration assessment, or outcome prediction was performed in this cross-sectional dataset. Therefore, the present findings should not be interpreted as evidence of clinical performance, but rather as an initial framework requiring validation in independent longitudinal cohorts.

The present analysis suggests that the stronger statistical influence of the renal domain in the original NCR-SI formulation is partly related to asymmetric score construction. Symmetric weighting schemes resulted in a more balanced distribution of domain contributions, highlighting that domain weighting represents a design choice rather than a strictly data-driven structural necessity. Future refinement of NCR-SI should therefore consider empirically calibrated weighting strategies.

### 4.2. Implications and Future Directions

Such descriptive mapping may be particularly useful in hypothesis-generating analyses of multimorbidity clustering within routine clinical datasets. Although not intended for clinical decision-making in its current form, such integrative approaches may support hypothesis generation regarding interactions between physiological systems that are traditionally assessed separately.

For example, NCR-SI may be used in research-oriented EMR datasets to descriptively identify patient clusters with convergent renal, metabolic, and psychosocial burden, thereby facilitating subgroup analyses, multimorbidity mapping, and hypothesis generation for outcome-based longitudinal studies.

Despite these limitations, this study provides foundational evidence that NCR-SI can serve as a useful descriptive tool for stratifying multimorbidity burden across neuro-psychic, metabolic, and renal dimensions. Future research should focus on validating NCR-SI across larger, more diverse populations, assessing its stability over time, and evaluating its association with clinically relevant outcomes, such as hospitalization, disease progression, and healthcare utilization. If validated longitudinally, NCR-SI is not intended to replace existing multimorbidity or risk indices, but rather to complement them by providing a transparent, domain-based summary of multimorbidity burden, including documented neuro-psychic involvement.

## 5. Conclusions

NCR-SI values showed a graded distribution across the three clinical context groups, with higher composite scores predominantly observed in the renometabolic context, intermediate values in the cardiometabolic context, and lower scores in the heterogeneous comorbidity group. These differences reflect the aggregation of domain-specific burdens captured by the NCR-SI structure rather than isolated abnormalities in individual biomarkers. Correlation analyses supported the internal coherence of the index, showing alignment between each NCR-SI domain and its corresponding clinical markers, while preserving partial independence of the neuro-psychic dimension. The minimal overlap with traditional comorbidity indices further supports the conceptual distinctiveness of NCR-SI as a physiology-oriented descriptive framework.

In summary, the Neuro–Cardio–Renal Stress Index (NCR-SI) provides a structured, descriptive approach for integrating neuro-psychic, metabolic, and renal dimensions of multimorbidity into a unified neuro–cardio–renal stress representation using real-world EMR data.

## Figures and Tables

**Table 1:** Descriptive comparison of renal and metabolic biomarkers across groups. Kruskal–Wallis *p*-values are reported for descriptive context only and are not intended for inferential interpretation, as group definitions are partly based on these same variables.

Variable	Mean (Group 1)	Mean (Group 2)	Mean (Group 3)	Test	*p*-Value
eGFR (mL/min/1.73 m^2^)	88.62	53.21	76.80	Kruskal–Wallis	1.95 × 10^−11^
UACR (mg/g)	17.32	32.09	45.74	Kruskal–Wallis	0.150
Serum creatinine (mg/dL)	0.84	1.07	0.96	Kruskal–Wallis	0.0078
TyG index	9.14	9.16	8.73	Kruskal–Wallis	0.0236
Number of medications	5.87	7.07	5.08	Kruskal–Wallis	0.00072

Group comparisons are presented for descriptive background only. *p*-values reflect expected differences inherent to cohort stratification and were not interpreted as independent outcome findings.

**Table 2:** Distribution of NCR-SI categories across the three groups.

Groups	Median NCR-SI	IQR	Low	Moderate	High
1—Cardiometabolic	5	4–5	16	30	1
2—Renometabolic	6	5–7	16	56	9
3—Other comorbidities	3.5	3–4	10	2	1

**Table 3:** Spearman correlation coefficients between the total NCR-SI score and individual domains under original and symmetric weighting schemes.

Model	*p* Total-R	*p* Total-C	*p* Total-N
original	0.755	0.533	0.509
symmetric	0.681	0.601	0.587

**Table 4:** Association between NCR-SI and Charlson Comorbidity Index.

Variables	Spearman ρ	*p*-Value	N
NCR-SI vs. CCI (age-adjusted)	0.085	0.311	143
NCR-SI vs. CCI (unadjusted)	0.172	0.040	143

**Table 5:** Spearman correlation coefficients between NCR-SI, its domains, and key biomarkers.

Variable	NCR-SI	N Domain	C Domain	R Domain	eGFR	UACR	TyG
NCR-SI	1.000	0.509	0.533	0.755	−0.709	0.202	0.416
N (Neuro)	0.509	1.000	0.140	0.085	0.024	0.095	−0.025
C (Cardiometabolic)	0.533	0.140	1.000	0.061	0.017	0.009	0.785
R (Renal)	0.755	0.085	0.061	1.000	−0.973	0.207	0.052
eGFR	−0.709	0.024	0.017	−0.973	1.000	−0.159	−0.076
UACR	0.202	0.095	0.009	0.207	−0.159	1.000	0.165
TyG	0.416	−0.025	0.785	0.052	−0.076	0.165	1.000

NCR-SI, Neuro–Cardio–Renal Stress Index; N domain, neuro-psychic domain; C domain, cardiometabolic domain; R domain, renal domain; eGFR, estimated glomerular filtration rate calculated using the MDRD equation; UACR, urinary albumin-to-creatinine ratio; TyG, triglyceride–glucose index. Correlation coefficients are Spearman’s rho (ρ).

## Data Availability

The data presented in this study are available on request from the corresponding author. The data are not publicly available due to privacy or ethical restrictions.

## Abbreviations

The following abbreviations are used in this manuscript:

CKD	Chronic kidney disease
C domain	Cardiometabolic domain of NCR-SI
eGFR	Estimated glomerular filtration rate
N domain	Neuro-psychic domain of NCR-SI
NCR-SI	Neuro–Cardio–Renal Stress Index
R domain	Renal domain of NCR-SI
SD	Standard deviation
SPSS	Statistical Package for the Social Sciences
TyG index	Triglyceride–glucose index
UACR	Urinary albumin-to-creatinine ratio
